# Nuclear Receptor CAR Represses TNFα-Induced Cell Death by Interacting with the Anti-Apoptotic GADD45B

**DOI:** 10.1371/journal.pone.0010121

**Published:** 2010-04-12

**Authors:** Yukio Yamamoto, Rick Moore, Richard A. Flavell, Binfeng Lu, Masahiko Negishi

**Affiliations:** 1 Laboratory of Reproductive and Developmental Toxicology, National Institute of Environmental Health Sciences, National Institutes of Health, Research Triangle Park, North Carolina, United States of America; 2 Section of Immunobiology, Yale University School of Medicine, New Haven, Connecticut, United States of America; 3 Department of Immunology, University of Pittsburgh, School of Medicine, Pittsburgh, Pennsylvania, United States of America; Dr. Margarete Fischer-Bosch Institute of Clinical Pharmacology, Germany

## Abstract

**Background:**

Phenobarbital (PB) is the most well-known among numerous non-genotoxic carcinogens that cause the development of hepatocellular carcinoma (HCC). PB activates nuclear xenobiotic receptor Constitutive Active/Androstane Receptor (CAR; NR1I3) and this activation is shown to determine PB promotion of HCC in mice. The molecular mechanism of CAR-mediated tumor promotion, however, remains elusive at the present time. Here we have identified Growth Arrest and DNA Damage-inducible 45β (GADD45B) as a novel CAR target, through which CAR represses cell death.

**Methodology/Principal Findings:**

PB activation of nuclear xenobiotic receptor CAR is found to induce the *Gadd45b* gene in mouse liver throughout the development of HCC as well as in liver tumors. Given the known function of GADD45B as a factor that represses Mitogen-activated protein Kinase Kinase 7 - c-Jun N-terminal Kinase (MKK7-JNK) pathway-mediated apoptosis, we have now demonstrated that CAR interacts with GADD45B to repress Tumor Necrosis Factor α ( TNFα)-induced JNK1 phosphorylation as well as cell death. Primary hepatocytes, prepared from *Car^+/+^, Car^−/−^, Gadd45b^+/+^* and *Gadd45b^−/−^* mice, were treated with TNFα and Actinomycin D to induce phosphorylation of JNK1 and cell death. Co-treatment with the CAR activating ligand TCPOBOP (1,4 bis[2-(3,5-dichloropyridyloxy)]benzene) has resulted in repression of both phosphorylation and cell death in the primary hepatocytes from *Car^+/+^* but not *Car^−/−^*mice. Repression by TCPOBOP was not observed in those prepared from *Gadd45b^−/−^* mice. *In vitro* protein-protein interaction and phosphorylation assays have revealed that CAR interacts with MKK7 and represses the MKK7-mediated phosphorylation of JNK1.

**Conclusions/Significance:**

CAR can form a protein complex with GADD45B, through which CAR represses MKK7-mediated phosphorylation of JNK1. In addition to activating the *Gadd45b* gene, CAR may repress death of mouse primary hepatocytes by forming a GADD45B complex and repressing MKK7-mediated phosphorylation of JNK1. The present finding that CAR can repress cell death via its interaction with GADD45B provides an insight for further investigations into the CAR-regulated molecular mechanism by which PB promotes development of HCC.

## Introduction

Nuclear receptor CAR was originally characterized as a PB-activated transcription factor that up-regulates the *cytochrome P4502B* genes [1], [2]. Soon after this, CAR became established as the nuclear receptor that regulates hepatic drug metabolism and excretion by coordinately activating various hepatic genes that encode cytochrome P450s, conjugation enzymes such as UDP-glucuronosyltransferases and sulfotransferases and drug transporters [3], [4], [5]. Recent work has extended the biological function of CAR far beyond the regulation of drug metabolism: for example, CAR is now implicated in the regulation of hepatic energy metabolism by cross talking with insulin or glucagon response transcription factors; FoxO1, FoxA2, PGC-1 and CREB [6], [7], [8], [9]. CAR is also found to play an essential role in nongenotoxic carcinogenesis; drug activation of CAR by PB resulted in the promotion of HCC development in mice, while no such promotion occurred in the absence of CAR [10], [11]. Thus, CAR has been suggested to regulate a cellular signal leading to cell growth and death.

GADD45B is a signal molecule inducible to response through external stimuli such as oxidative stress, inflammation and UV irradiation [12], [13], [14]. GADD45B is an anti-apoptotic factor that directly binds to MKK7 and inhibits MKK7-dependent phosphorylation of JNK1/2 to repress apoptosis [15], [16] and, moreover, a possible role of GADD45B in hepatocarcinogenesis and hepatocyte proliferation has been suggested [17], [18]. To search for a CAR-regulated signal molecule that may be involved in promotion of HCC development, we hypothesized that this factor should exhibit CAR-dependent induction by nongenotoxic carcinogens long before the liver develops tumors and should be further induced in tumor tissues. c-JUN, MDM2 and FoxM1B were previously suggested as signal molecules critical for HCC development [10], [19], [20]. While none of these genes fit with this hypothesis, the expression pattern of the *Gadd45b* gene is consistent with the hypothesis. In addition, the *Gadd45b* gene has been known to be induced after short-term treatment with CAR activator TCPOBOP in mouse livers [21], [22]. Therefore, here we have further investigated GADD45B as a candidate of CAR-regulated signal molecule.

For present investigations, we have used mouse primary hepatocytes prepared from *Car^+/+^*, *Car^−/−^*, *Gadd45b^+/+^* and *Gadd45b^−/−^* mice. Cell death was induced by treatment of primary hepatocytes with TNFα and actinomycin D (ActD) in the presence or absence of TCPOBOP to examine whether or not CAR could repress cell death and as to whether GADD45B could mediate the CAR-mediated repression of cell death. Moreover, GST-pull down and co-immunoprecipitation assays were performed to characterize the binding nature of CAR to GADD45B. Given the finding that TCPOBOP treatment decreased the phosphorylation of JNK in the primary hepatocytes from *Car^+/+^* but not from *Car^−/−^*, *in vitro* kinase assays were employed to demonstrate CAR potentiating GADD45B-dependent inhibition of JNK1 phosphorylation by MKK7. Here we present the experimental considerations consistent with the conclusion that CAR represses the death of TNFα-induced primary hepatocytes by increasing GADD45B activity and inhibiting the MKK7-dependent phosphorylation of JNK.

## Results and Discussion

### CAR repressing cell death

**Figure 1:**
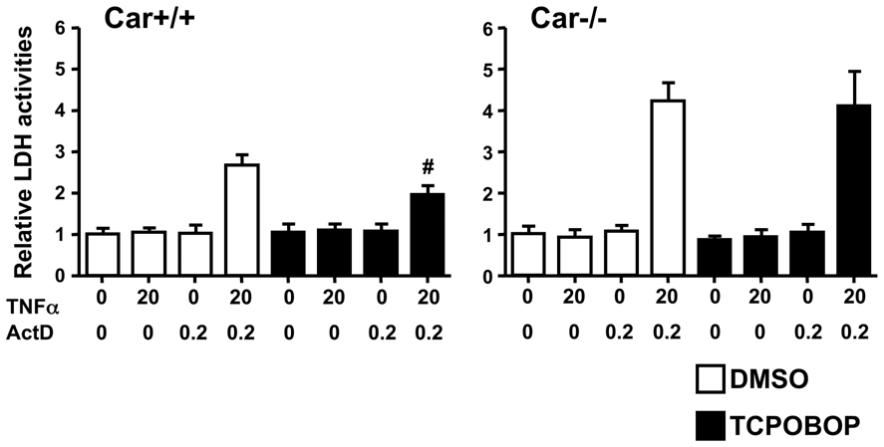
CAR-mediated repression of cell death. Approximately the same number (1×10^5^) of primary hepatocytes prepared from the livers of *Car^+/+^* and *Car^−/−^* mice were plated on plastic dishes (24 wells). Cells were pretreated with DMSO or 250 nM TCPOBOP for 24 hours and were subsequently treated with or without TNFα (20 ng/ml) plus ActD (0.2 µg/ml) for 16 hours. Release of lactate hydrogenase (LDH) in culture media was determined as described in the Materials and Methods. Each value represents the mean +/− S.D as fold changes relative to DMSO without TNFα and ActD, which was independently reproduced 3 times. Symbol # indicates statistical significance between DMSO-pretreated cells and TCPOBOP-pretreated cells (p<0.05).

To examine whether or not CAR could play a role in cell death, mouse primary hepatocytes were treated with either TNFα or ActD or were co- treated with both TNFα and ActD to induce cell death. TNFα is known to induce apoptosis of hepatocytes [23], [24]. In our experimental conditions, only the co-treatment of TNFα and ActD increased the cell death of primary hepatocytes prepared from *Car^+/+^* mice by approximately 3-fold (Figure 1). Pre-treatment with CAR activator TCPOBOP resulted in an approximately 55% reduction of the TNFα/ActD induced cell death. In the primary hepatocytes prepared from *Car^−/−^* mice, the TNFα/ActD induced cell death was already elevated approximately 1.5-fold over the corresponding cell death of *Car^+/+^* primary hepatocytes in the absence of TCPOBOP (Figure 1). Consistent with the lack of CAR, TCPOBPO treatment did not affect the cell death sensitivity of *Car^−/−^* primary hepatocytes. The results indicated that CAR repressed the cell death: TCPOBOP activation of CAR attenuated the TNFα/ActD induced cell death of mouse primary hepatocytes and that cell death was intrinsically higher in *Car^−/−^* primary hepatocytes.

### *Gadd45b* as the CAR-regulated gene

**Figure 2:**
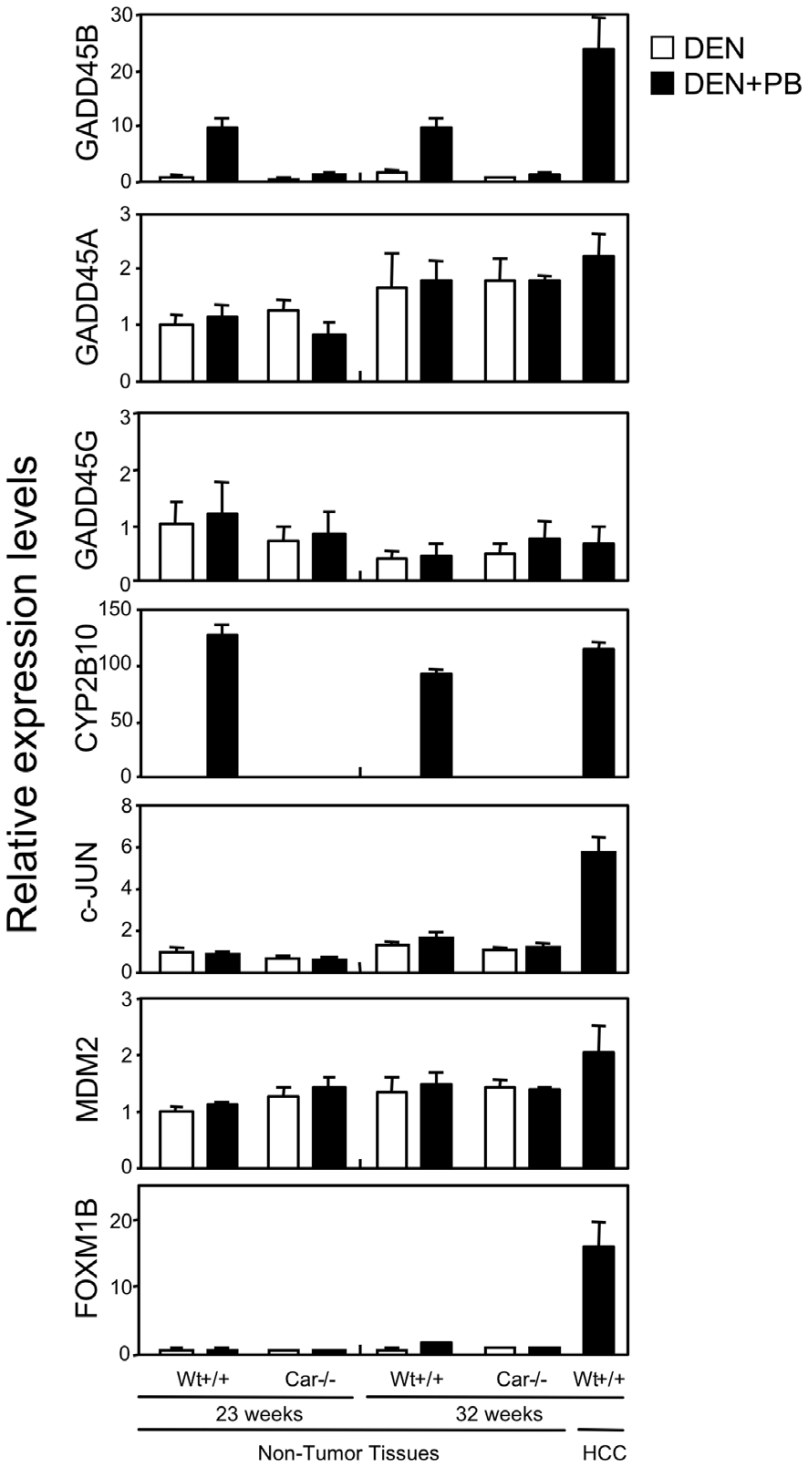
Screening of *Gadd45b* for a CAR-regulated gene in chronic PB-treated tissues. RNAs were prepared from non-tumor tissues of the PB-treated livers for 23- and 32- weeks and from tumor tissues of the PB-treated livers for 32 weeks. At least, six animals were used for each group. Each value represents the mean +/− S.D as fold changes relative to the RNAs from the non-tumor liver tissues of wild type mice treated with diethylnitrosamine (DEN) for 23 weeks.

Given the fact that CAR repressed cell death of mouse hepatocytes, we searched for a CAR-regulated gene that encodes a signal molecule involved in cell death using the liver samples collected during our previous studies of PB promotion of HCC development in the *Car^+/+^* and *Car^−/−^* mice [11]. For the search, two criteria were placed: the CAR-regulated gene should be induced by PB before the liver developed HCC and this gene should be continuously induced further in the tumor tissues. The *Gadd45b* gene was reported to be induced by a 24 hr treatment with TCPOBOP in the mouse liver [21]. The expression of the *Gadd45b* gene was determined in the liver samples of the *Car^+/+^* and *Car^−/−^* treated with PB for 23 weeks and for 32 weeks. No tumors developed in the livers treated 23 weeks. From the livers treated 32 weeks, tumor tissues and the non tumor tissues were separately dissected out for analysis. Real-time PCR assays showed that GADD45B mRNA had increased in the 23 weeks of PB treatment in the livers of the *Car^+/+^* mice but not of *Car^−/−^* mice (Figure 2). This mRNA was further increased in the tumor tissues. Conversely, the other members of the GADD45 family, GADD45A and GADD45G mRNAs were not induced by PB treatment.

The two signal molecules c-JUN and FOXM1B have been suggested as being the factors responsible for HCC development in PB-treated mouse liver based on the fact that the *c-Jun^−/−^* and *Foxm1b^−/−^* mice exhibited attenuated HCC development [19], [20]. In addition, MDM2 was implicated as the CAR-regulated gene that was important for PB induced HCC development [10]. Real time PCR was performed to determine the expression of these three genes. Neither c-JUN, FOXM1B nor MDM2 mRNA were induced by PB either for 23 or 32 weeks treatments, although they were up-regulated in the tumor tissues, c-JUN and FOXM1B in particular (Figure 2). Thus, none of these three genes appeared to be regulated by CAR, implying that they are not directly responsible for CAR-mediated tumor promotion by PB, although they are critical for maintaining tumor status. Therefore, we chose GADD45B to further investigate its potential at being the CAR-regulated signal molecule that may regulate the TNFα-dependent cell death of mouse primary hepatocytes.

### CAR repressing JNK phosphorylation in mouse primary hepatocytes

**Figure 3:**
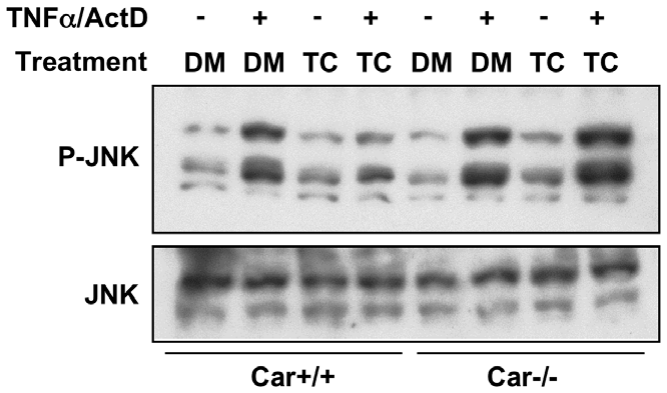
CAR-mediated repression of JNK phosphorylation. Primary hepatocytes (1×10^5^ cells/well) were prepared from *Car^+/+^* and *Car^−/−^* mice, were pretreated with either DMSO (DM) or TCPOBOP (TC; 250 nM) and were co-treated with TNFα and ActD to induce cell death. Subsequently cell extracts were prepared for Western blot analysis for phosphorylated JNK (P-JNK) and total JNK.

GADD45B is known to directly bind to MKK7 inhibiting its activity to phosphorylate JNK, resulting in the repression of apoptosis [16], [25]. Based on the finding that CAR up-regulated the *Gadd45b* gene, we examined whether or not the activation of CAR could down-regulate JNK phosphorylation in mouse primary hepatocytes (Figure 3). TCPOBOP treatment attenuated JNK phosphorylation by TNFα/ActD in the primary hepatocytes of the *Car^+/+^* but not *Car^−/−^* mice. The levels of JNK protein remained unchanged by TCPOBOP treatment in all primary hepatocytes. Although the effect was not as large as that observed with the cell death assays (Figure 1), the JNK phosphorylation level was higher in the DMSO-treated *Car^−/−^* hepatocytes when compared with the corresponding *Car^+/+^* hepatocytes. Thus, CAR was clearly capable of attenuating the phosphorylation of JNK in mouse primary hepatocytes, tempting us to investigate the molecular mechanism of CAR-mediated repression of JNK phosphorylation.

### CAR potentiating GADD45B-mediated inhibition of MKK7 activity

**Figure 4:**
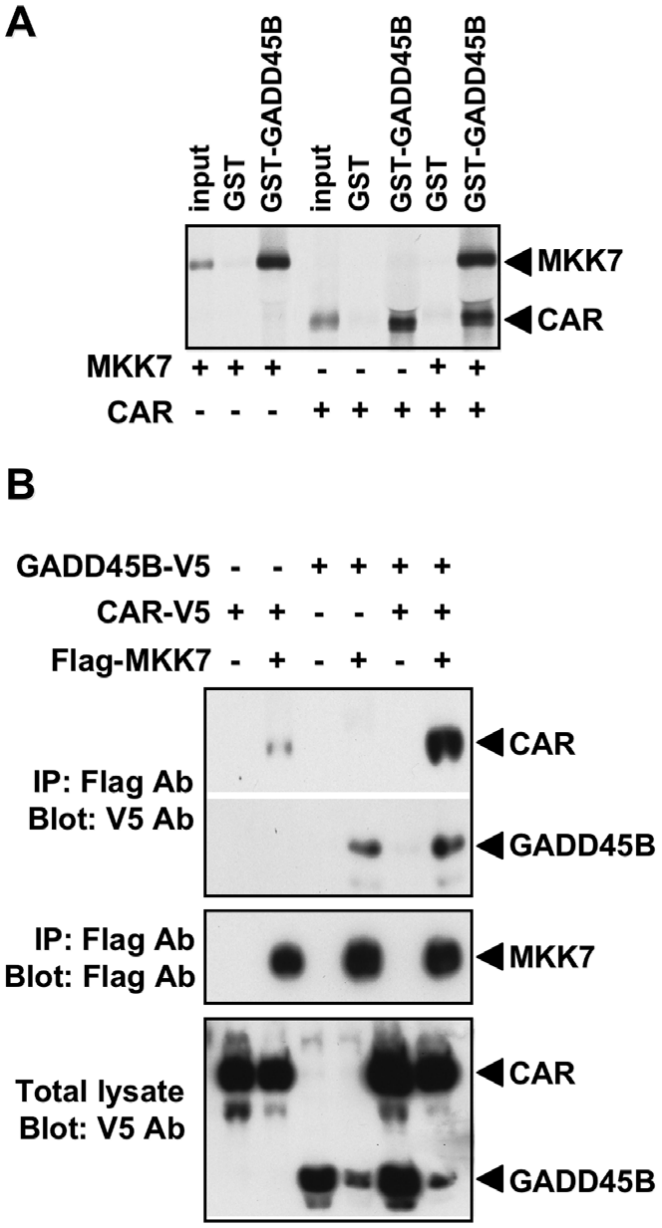
GADD45B facilitating the ability of CAR to form a complex with MKK7. A. GST pull down assays showing direct binding of GADD45B to CAR and MKK7. GST-GADD45B was incubated with the *in vitro* translated MKK7 and/or CAR, which was subjected for SDS polyacrylamide gel analysis as described in the Materials and Methods. B. Co-immunoprecipitation (Co-IP) assays to show formation of a CAR complex with MKK7. CAR-V5 was co-expressed with Flag-MMK7 in the presence and absence of GADD45B-V5 in HEK293T cells, co-precipitated by anti-Flag antibody and was analyzed by Western blot using anti-V5 and anti-Flag antibodies.

Confirming previous observations [15], [16], GST-pull down assays showed the binding of GADD45B to MKK7 (Figure 4A). We also found that GADD45B binds directly to CAR. Subsequently, the interactions between CAR, GADD45B and MKK7 were examined by co-expressing combinations of GADD45B-V5, CAR-V5 and Flag-MKK7 in HEK293T cells and by co-immunoprecipitating the GADD45B and CAR with anti-Flag antibody from the cell extracts. Flag antibody precipitated MKK7 as shown by the Western blot with anti-Flag antibody and co-precipitated GADD45B-V5 when it was co-expressed with Flag-MKK7 (Figure 4B). CAR-V5 was also co-precipitated by Flag antibody, suggesting that CAR could form a complex with MKK7. With co-expression of all three proteins, CAR-V5 profoundly increased its binding to Flag-MKK7 in the presence of GADD45B-V5, whereas GADD45B binding to MKK7 remained unchanged (Figure 4B). These results indicated that GADD45B could increase the formation of a CAR-MKK7 complex. This complex might be a triple complex of CAR-GADD45B-MKK7, in which the molecular ratio of three proteins remained to be determined by future investigations.

**Figure 5:**
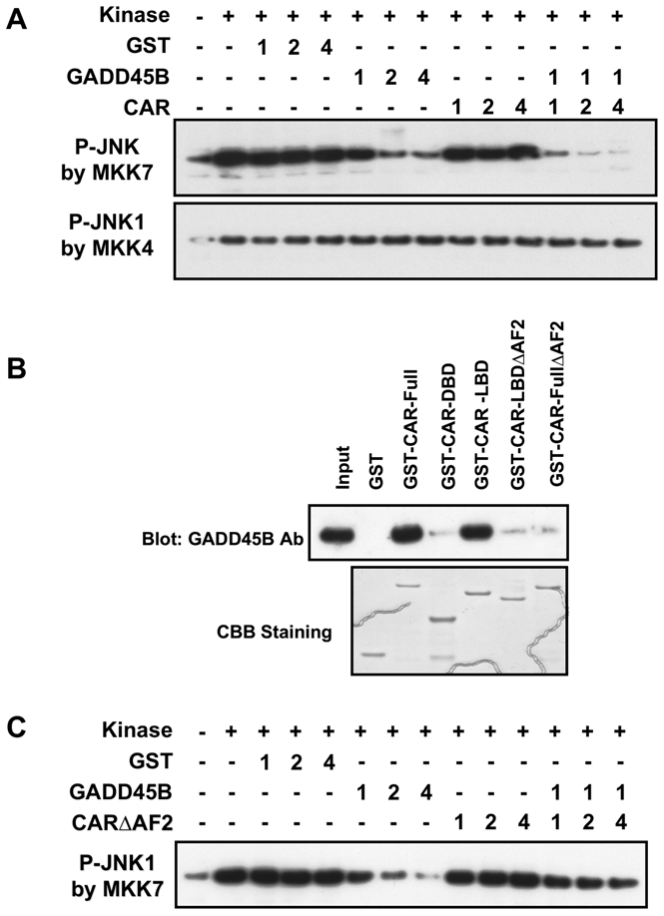
CAR potentiating the ability of GADD45B to inhibit MKK7 activity. A. GST-CAR was pre-incubated with recombinant GADD45B and the active kinase MKK7 or MKK4 for 20 min at room temperature. Kinase reactions were initiated by adding ATP and purified substrate JNK1. Following incubation for 30 min at room temperature, incubation mixtures were applied on a SDS gel and were subjected to Western blotting analysis using anti-phospho-JNK antibody. Arabic numbers indicate the µg amounts of recombinant proteins in pre-reaction mixtures: 1 means approximately 1 µg of protein. B. CAR required its AF2 domain for the inhibitory activity. GST-pull down assays shows AF2 domain of CAR was required to bind to GADD45B. Purified GST-CAR mutants were incubated with purified GADD45B protein. Western blotting analysis was performed using anti-GADD45B antibody to detect binding. C. MKK7 kinase assays showing requirement of the AF2 domain for inhibition. The GST-CARΔAF2 mutant was used to measure its inhibitory activity of MKK7 kinase, which was analyzed Western blotting using anti-Phospho-JNK antibody as described in Experimental Procedures.

To examine whether or not forming a complex with CAR affects MKK7 kinase ability to phosphorylate JNK1, an *in vitro* assay system of the MKK7-dependent JNK phosphorylation was reconstituted using purified active MKK7 and substrate JNK1 (Figure 5A). As expected, the JNK phosphorylation was inhibited in a concentration-dependent manner by addition of GADD45B to the reaction mixture. CAR had no effect on the levels of JNK phosphorylation at any concentrations tested. However, in the presence of CAR, GADD45B greatly enhanced its inhibition of JNK1 phosphorylation by MKK7 (Figure 5A). The JNK1 phosphorylation by MKK4 was not affected by GADD45B, CAR or GADD45B plus CAR, serving as a negative control for the specific GADD45B-MKK7 reaction. Thus, these results were consistent with the hypothesis that CAR, by forming a complex with GADD45B and MKK7, strongly potentiated GADD45B-dependent inhibition of MKK7-mediated phosphorylation of JNK1. Moreover, CAR required the AF2 domain for binding to GADD45B in GST-pull down assays (Figure 5B) and the CAR lacking the AF2 domain was not capable of potentiating the GADD45B-mediated inhibition of JNK1 phosphorylation by MKK7 (Figure 5C).

### CAR not repressing cell death in the absence of GADD45B

**Figure 6:**
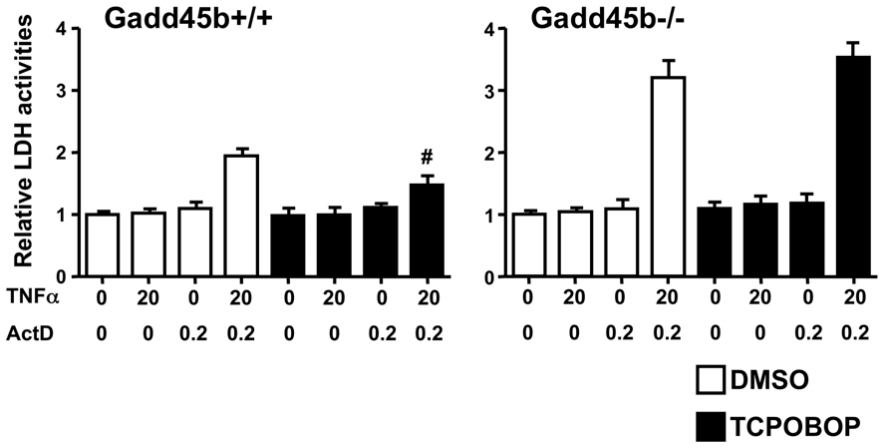
No CAR-dependent repression of cell death in the absence of GADD45B. Primary hepatocytes were prepared from *Gadd45b^+/+^* and *Gadd45b^−/−^* mice and were subjected to cell death assays as described in Materials and Methods and in the legend of Figure 1. Each value represents the mean +/− S.D as fold changes relative to DMSO without TNFα and ActD. The data shown reproduced in 3 independent experiments. Symbol # indicates statistical significance between DMSO-pretreated cells and TCPOBOP-pretreated cells (p<0.05).

Given the fact that CAR inhibited MKK7 activity via its interaction with GADD45B, we hypothesized that CAR may require the presence of GADD45B to repress cell death. To test this hypothesis, we performed cell death assays with mouse primary hepatocytes prepared from *Gadd45b^+/+^* and *Gadd45b^−/−^* mice. Co-treatment with TNFα and ActD induced the cell death of the *Gadd45b^+/+^* hepatocytes approximately 2-fold (Figure 6). This rate of induction was smaller than that observed with the *Car^−/−^* hepatocytes (Figure 1), which appeared to be due to the fact that these cells originate from different strains with different sensitivities to TNFα and ActD: the genetic backgrounds of *Gadd45b^−/−^* and *Car^−/−^* mice are C57BL/6 and C3H, respectively. Similar to the cell death observed with *Car^+/+^* hepatocytes (Figure 1), TCPOBOP activation of CAR attenuated the cell death approximately 45%. The cell death of the *Gadd45b^−/−^* hepatocytes was significantly elevated in the absence of TCPOBOP and was not attenuated by TCPOBOP treatment (Figure 6). These cell death responses of the *Gadd45b^−/−^* hepatocytes were reminiscent of those with the *Car^−/−^* hepatocytes. Thus, CAR could not repress the TNFα/ActD induced cell death in the absence of GADD45B.

Using mouse primary hepatocyetes from *Car^−/−^* and *Gadd45b^−/−^* mice, here we have determined the molecular mechanism by which CAR represses TNFα-induced cell death through its interactions with GADD45B. GADD45B is an anti-apoptotic factor that directly interacts with MKK7 and inhibits MKK7-dependent phosphorylation of JNK to repress apoptosis. CAR can increase GADD45B-dependent inhibition of the MKK7 kinase activity by transcriptional up-regulation of the *Gadd45b* gene as well as by directly binding to GADD45B, thereby potentiating its inhibitory activity and repressing TNFα-induced cell death in the *Gadd45b^−/−^* primary hepatocytes. Various types of crosstalk between signal transduction pathway and nuclear receptor have been reported. For example, to control various cellular responses, nuclear receptors regulate gene transcription by acting as cofactors (*e.g.* GR to AP1, CAR to Fox proteins) [7], [8], [26]. In addition to genomic regulation, several nuclear steroid hormone receptors exhibit non-genomic function to regulate cell signaling; ERα associates with the epidermal growth factor receptor mediated-signaling to activate extracellular signal-regulated kinase [27]. Compared with these known mechanisms, the direct interaction of CAR with GADD45B to potentiate the anti-apoptotic activity of GADD45B is a unique mechanism.

Many CAR activators such as PB and TCPOBOP are non-genotoxic carcinogens and CAR itself has been implicated as being the determining factor in PB/TCPOBOP promotion of HCC development in mice [10], [11]. Tumor promotion considers the pathological incidence in which non-genotoxic carcinogens, such as chronic PB treatment, determine tumor development in organs pre-initiated by genotoxic carcinogens such as diethylnitrosamine [28]. The pathology of tumor promotion is not well defined and its molecular mechanism is virtually unknown at the present time. However, PB must promote HCC development through activation of a CAR-regulated gene since CAR is a target of PB action and because PB does not promote HCC development in the absence of CAR. In this respect, previously suggested c-JUN, FOXM1B and MDM2 do not appear to be the critical factor responsible for PB promotion since these genes are not regulated by CAR in our experimental system. The question is whether or not CAR-mediated repression of apoptosis through the GADD45B-MKK7-JNK pathway is, in fact, responsible for PB promotion of HCC development. We determined caspase-3 positive hepatocytes of the mouse livers during PB promotion (unpublished observation). No PB- and CAR-dependent increase of apoptosis was detected. Thus, not only the role of the GADD45B pathway but also that of general apoptosis in PB tumor promotion remains elusive. Now, future tumor promotion studies using *Gadd45b^−/−^* and *Jnk^−/−^* mice may provide us with clues to understand the molecule mechanism of how CAR regulates PB promotion of HCC development.

## Materials and Methods

### Materials

Phenobarbital, TCPOBOP, anti-Flag M2 antibody and anti-Flag M2-agarose were purchased from Sigma-Aldrich; recombinant mouse TNFα from R&D systems; actinomycin D from Biomol Reserch Labs., Inc; William's medium E, penicillin, streptomycin, L-glutamate, HEPES, anti-V5 antibody, HepatoZYME-SFM and Superscript First-Strand Synthesis System from Invitrogen; fetal bovine serum from Atlanta biological; anti-phospho-SAPK/JNK (Thr183/Thy185) antibody and anti-SAPK/JNK (56G8) antibody from Cell Signaling Technology; GADD45B (N-19) antibody from Santa Cruz Biotechnology; CytoTox 96 kit from Promega; Complete mini protease inhibitor cocktail tablet from Roche. All other reagents were purchased from Sigma, unless indicated otherwise.

### Animals

*Car* ^−/−^ and *Gadd45b* ^−/−^ mice were previously generated and characterized [11], [29]. *Car* ^−/−^ mice used in these studies were maintained in C3H genetic background. *Gadd45b* ^−/−^ mice used in these studies were maintained in C57BL/6 genetic background. All animals were housed in a temperature-controlled environment with 12 hr light/dark cycles with access to standard chow and water *ad libitum*. Age-matched groups of 8 to 12-week-old mice were used for preparation of primary hepatocytes. All protocols and procedures were approved by the National Institutes of Health Animal Care and Use Committee and were in accordance with National Institutes of Health guidelines.

### Mouse primary hepatocytes

Mouse primary hepatocytes were prepared from male mice by a two-step collagenase perfusion method and cultured as previously described [1]. Hepatocytes were suspended in Williams medium E supplemented with 7% fetal bovine serum, 1 X Liquid Media Supplement (5 µg/ml insulin, 5 µg/ml transferrin, 5 µg/ml selenite; Sigma-Aldrich), 2 mM L-glutamine and 30 mM pyruvate and were allowed to adhere to 24-well plates (1×10^5^ cells) for 4 hours in a CO2 incubator at 37°C. These hepatocytes were maintained in HepatoZYME-SFM for 16 h and were subsequently pretreated with DMSO or 250 nM TCPOBOP for 24 hours. To induce cell death, hepatocytes were incubated with or without 20 ng/ml TNFα plus 200 ng/ml Actinomycin D in HepatoZYME-SFM for 16 hours. Release of lactate dehydrogenase (LDH) was determined in the supernatant of hepatocyte cultures using the CytoTox 96 kit according to the manufacturer's recommendations. DMSO treated cells were used to normalize the results. Significant differences were analyzed by student t-test.

### Western blotting

Protein was separated by SDS-PAGE and transferred to PVDF membrane (Immobilon P, Millipore). The membranes were probed with specified primary antibodies; anti-phospho-SAPK/JNK (Thr183/Thy185) antibody and anti-SAPK/JNK (56G8) antibody (Cell Signaling Technology), anti-GADD45B (N-19) antibody (Santa Cruz Biotechnology), anti-Flag antibody (Sigma-Aldrich) and anti-V5 antibody (Invitrogen).

### Real time PCR

All Real Time PCR data were obtained using RNA isolated from tissues of individual animals. Total RNAs were isolated from mouse hepatic tissues using the RNeasy (QIAGEN), from which cDNAs were synthesized from total RNAs with the Superscript First-Strand Synthesis System and random hexamer primers. Real Time PCR measurements of individual cDNAs were performed with the ABI prism 7700 sequence detection system. Gene-specific primers and probes were purchased as pre-designed TaqMan Gene Expression Assays gene-specific probe and primers mixture (PE Applied Biosystems). Assay ID number of pre-designed TaqMan Gene expression Assay (gene, assay ID number) used in this study were as follows: Gadd45b, Mm00435123_m1; Gadd45a, Mm00432802_m1; Gadd45g, Mm00442225_m1; Cyp2b10, Mm00456591_m1; c-Jun, Mm00495062_s1; Mdm2, Mm00487656_m1; Foxm1b, Mm00514924_m1. The TaqMan rodent GAPDH control reagent (PE Applied Biosystems) was used as an internal control.

### GST-pull down

GST pull-down assays with *in vitro* translated proteins or purified proteins were performed as previously reported [30]. Glutathione S-transferase (GST) fusion proteins, GST-GADD45B, GST-CAR, GST-CAR-DBD, GST-CAR-LBD, GST-CAR-LBDΔAF2, GST-CARΔAF2 were expressed in Escherichia coli strain BL21 cells and purified with glutathione-Sepharose 4B (Amersham Biosciences). MKK7 and CAR were labeled with [35S] methionine using the TNT T7 quick-coupled transcription/translation system (Promega). GST-GADD45B protein was purified with glutathione-Sepharose 4B and digested with thrombin (Sigma-Aldrich). Bound proteins were detected by autoradiography after SDS-PAGE separation for MKK7 and CAR. GADD45B proteins were detected by Western blotting using GADD45B (N-19) antibody (Santa Cruz Biotechnology). Western blots with similar results were reproduced twice.

### Immunoprecipitation

HEK293T cells were transfected with Flag-tagged MKK7 and V5-tagged CAR and/or V5-tagged GADD45B for 16 hr, then were rinsed once with Tris-buffered saline (TBS) and lysed with ice-cold buffer (50 mM Tris-HCl, pH 7.5 containing 150 mM NaCl, 1 mM EDTA, 1 mM dithiothreitol, 1% Triton X-100, 0.5% NP40, and Complete mini protease inhibitor cocktail tablet). Resulting cell lysates were sonicated and centrifuged for 20 min at 20,000×g at 4°C. The supernatants were incubated with anti-Flag M2-agarose for 1 h at 4°C. The agarose were washed with ice-cold buffer (50 mM Tris-HCl, pH 7.5 containing 150 mM NaCl, 1 mM EDTA, 1 mM dithiothreitol, 0.4% Triton X-100, 0.2% NP40 and Complete mini protease inhibitor cocktail tablet), from which bound proteins were eluted in LDS (lithium dodecyl sulfate) sample buffer (Invitrogen) and analyzed by SDS-PAGE and western blotting with anti-Flag or anti-V5 antibody. Western blots with similar results were reproduced 3 times.

### *In vitro* kinase assays

GST-JNK1, GST-GADD45B, GST-CAR, GST-CARΔAF2 and GST were expressed in *Escherichia coli* BL21 cells and purified using glutathione-Sepharose 4B. GST-JNK1, GST-GADD45B, GST-CAR and GST-CARΔAF2 proteins were excised by thrombin. Flag-MKK7 and Flag-MKK4 were purified from HEK293T cells by using anti-Flag M2-agarose. Given GST-proteins were pre-incubated in a kinase buffer containing 25 mM Tris-HCl, pH 7.5, 5 mM β-glycerolphosphate, 0.1 mM Na_3_VO_4_, 10 mM MgCl_2_, 1 mM dithiothreitol for 20 min at room temperature. Kinase reaction was the initiated by adding GST-JNK1 as kinase substrate and 200 µM ATP and was continued for 30 min at room temperature. The reaction mixtures were subjected to SDS-PAGE and to Western blotting analysis using anti-phospho-JNK antibody. Western blots with similar results were independently reproduced 3 times.
